# Consolidating strategic information to monitor progress against the UNAIDS 90–90–90 targets: evaluating the operational feasibility of an electronic HIV testing register in Cape Town, South Africa

**DOI:** 10.1186/s12913-020-05517-7

**Published:** 2020-08-06

**Authors:** Nisha Jacob, Brian Rice, Emma Kalk, Alexa Heekes, Jennie Morgan, Samantha Brinkmann, James Hargreaves, Marsha Orgill, Andrew Boulle

**Affiliations:** 1 School of Public Health and Family Medicine, Faculty of Health Sciences, University of Cape Town, Cape Town, South Africa; 2 London School of Hygiene and Tropical Medicine, London, United Kingdom; 3 Western Cape Government: Health, Cape Town, South Africa

**Keywords:** Digitisation, HIV point-of-care testing, Case-based surveillance, Implementation, Health information systems

## Abstract

**Background:**

HIV diagnosis in South Africa is based on a point-of-care testing (PoCT) algorithm with paper-based record-keeping. Aggregated testing data are reported routinely. To facilitate improved HIV case-based surveillance, the Western Cape Province implemented a unique pilot intervention to digitise PoCT results, at an individual level, and generate an electronic register using the newly developed Provincial Health Data Centre (PHDC). We describe the intervention (phased) and present an evaluation of the operational feasibility of the intervention. We also offer implementation insights into establishing electronic capture of individual level testing data.

**Methods:**

Cross-sectional analyses were conducted on records of all patients attending a local Community Health Centre who had an HIV-PoCT during the study period. Data from the intervention were linked to the PHDC using a unique identifier and compared with aggregate data from the paper-based register. Correlation coefficients were calculated to quantify the correlation between the two monthly datasets. To support an understanding of the findings, the Department of Health project management team generated reflections on the implementation process, which were then grouped thematically into implementation lessons.

**Results:**

In total, 11,337 PoCT records were digitised (70% (7954) during Phase I; and 30% (3383) during Phase II). Linkage of forms to the PHDC was 96% in Phase I and 98% in Phase II. Comparison with aggregate data showed high correlation during Phase I, but notable divergence during Phase II. Divergence in Phase II was due to stringent data quality requirements and high clinical staff turnover. Factors supporting implementation success in Phase I included direct oversight of data capturing by a manager with clinical and operational insight. Implementation challenges included operational, health system, and high cost-related issues.

**Conclusions:**

We demonstrate that rapid digitisation of HIV PoCT data, without compromising currently collected aggregate data, is operationally feasible, and can contribute to person-level longitudinal HIV case-based surveillance. To take to scale, we will need to improve PoCT platforms and clerical and administrative systems. Although we highlight challenges, we demonstrate that electronic HIV testing registers can successfully replace manual registers and improve efforts to monitor and evaluate HIV testing strategies.

## Background

### Monitoring progress towards the 90–90-90 targets

The United Nation’s programme on HIV/AIDS (UNAIDS) set the 90–90-90 targets in 2015, aiming to diagnose 90% of those who are HIV infected, treat 90% of those who are diagnosed HIV positive and virally suppress 90% of those receiving antiretroviral treatment [1]. These targets are based on the World Health Organization (WHO) universal test and treat principle, also introduced in 2015 [2]. HIV case-based surveillance is a cost-effective method, recommended by WHO, to measure progress towards achieving the 90–90-90 targets [3]. Distinctive features of case-based surveillance are obtaining individual-level data for each person diagnosed and linking these data to other key data points along the HIV care cascade [3]. Such systems have been implemented in many low, middle and high-income countries [3, 4]. Many countries, particularly in high income settings, have also welcomed electronic health record keeping in the clinical environment [5], however this is not a viable option in various low-and-middle income contexts for several reasons, including complex infrastructure, network requirements and user resistance [6–8]. Despite the high burden of HIV in sub-Saharan Africa and the existence of routine HIV program monitoring, case-based surveillance has not been implemented fully [3]. Strategic information on HIV is frequently attained using parallel, costly donor-driven data collection processes due to weak health information systems [3, 9].

The first important steps to achieving the 90–90-90 targets is people living with undiagnosed HIV being diagnosed, and diagnoses both at the individual and population level being accurately monitored and measured [3]. Studies in India, Nigeria and Ethiopia have shown limited use of routine local data for health-system planning and decision-making [10, 11]. A situational assessment of data collection systems in Tanzania, Kenya and South Africa revealed several barriers to implementation of case-based surveillance, including multiple data collection systems, poor interoperability and inadequate capacity [3]. Many of these data collection systems are paper-based [3]. The validity of manual paper-based recorded patient level data which is later aggregated for reporting is difficult to assess, coupled with the administrative burden on clinical staff. Such data are known to be prone to error and double counting [12] and often lack the necessary detail required for active surveillance [13].

### Health information systems in South Africa

Health information systems can significantly support the health system at large, however health information systems are not universal across country contexts [3, 14]. In South Africa, TIER. Net is an HIV health information system which electronically captures longitudinal individual-level information on patients on antiretroviral *treatment* (ART) [3, 6]. However, HIV *testing* information is not captured on this system in many facilities, particularly information on those testing negative. Electronic linkage of individual-level patient data is possible in South Africa using a unique patient identifier e.g. patient folder number, but these identifiers have not been fully implemented in many provinces [15]. The Western Cape Provincial Government (one of South Africa’s nine provinces) has developed a Provincial Health Data Centre (PHDC) in which all individual-level routine data captured on electronic platforms in the province are consolidated on a single platform, leveraging the patient folder number as the unique patient identifier [15, 16]. This allows linkage of various information systems, including laboratory, pharmacy, and patient administration, providing a rich source of individuated health information, in the absence of routine electronic patient health records. Within this environment, disease-specific patient cascades may be developed using specific markers of care at different points in the treatment cascade [15]. The laboratory information system, governed by the National Health Laboratory Services (NHLS), is a key contributor of HIV surveillance data [17], as specific laboratory tests are indicative of key points within the HIV care continuum. These laboratory tests are conducted centrally under rigorous quality control processes. These central laboratory data are consolidated in the PHDC daily along with datasets from various other provincial data platforms. Consolidation of data on a single central platform supports integrated clinical care, whilst also facilitating more timely and comprehensive surveillance and further epidemiological and operational analyses [15].

### Point of care HIV testing in South Africa

While the PHDC platform supports health surveillance using routine data, an HIV case-based surveillance system requires individual level data on diagnosis of HIV [3]. An HIV diagnosis in South Africa is based on a point-of-care rapid-testing algorithm with paper-based record-keeping [3]. HIV testing is conducted primarily by HIV counsellors and nursing staff and information is entered into paper-based registers. Testing data are subsequently manually tallied, with aggregates reported to the Provincial Departments of Health on a monthly basis [3]. It is well-recognised that PoCT are less accurate than central laboratory tests as the tests are conducted by busy clinical staff members who are not trained rigorously on laboratory quality control and quality assurance [18, 19]. However, PoCT are favoured in high-burden, resource-limited settings as access to care is improved by more immediate availability of test results and linkage to further to care [18].

Monitoring and surveillance systems that are not part of an electronic information system cannot be linked to the PHDC. This is true for all point of care testing (PoCT) information in the province, which is not collected digitally. Although aggregate HIV testing data are reported electronically, individual testing data remain in paper-based registers and paper-based facility patient records. The lack of integration of PoCT results within the NHLS, and the broader PHDC, may hamper laboratory-informed case-based disease surveillance, HIV prevention planning, as well as limit information available to clinicians when evaluating historical laboratory investigations as part of clinical decision-making. In high-burden clinical environments, this results in longer, less efficient clinical consultations and unnecessary and costly repetition of PoCT [19]. Currently, the only electronically captured HIV test results within the NHLS are from enzyme-linked immunosorbent assay (ELISA) tests which are conducted at the central laboratory if rapid PoCT results are discordant. Negative HIV PoCT results are not captured electronically in any routine information system. In addition, the maintenance of the existing paper-based registers is onerous for busy clinical staff. Register reports are often delayed, and are rarely available to, or used by, the clinical staff who collect the data.

Various advanced PoCT devices are available which have the functionality to capture test results and patient information directly into electronic information systems [19]. Such devices are not widely used in the South African public health sector and may require significant resource and training investment before wide-scale implementation. Manual PoCT systems are thus likely to remain in the clinical setting.

There is currently a gap in literature on establishment of case-based surveillance systems in high-burden settings, including the digitisation of HIV PoCT [3, 12]. The maturation of the health information system in the Western Cape Province presented a unique opportunity to implement case-based surveillance through inclusion of routine individual-level electronic HIV testing data.

### Development of a pilot intervention to establish an electronic HIV testing register

Integration of PoCT results into an existing consolidated individual patient data environment was considered by provincial health managers, as it was likely to be more efficient and less onerous than developing a parallel system just for PoCT result digitisation. Given the close link between laboratory tests and PoCT and given that the NHLS is equipped with an efficient transport service that has daily contact with all provincial health facilities where PoCT is utilised, integration of PoCT into the NHLS was considered. Since NHLS is widely used by clinicians for retrieval of results, integration into the NHLS would further enhance accessibility of patient results. Furthermore, digitisation would enable the creation of actionable patient lists where linkage to care is delayed, thus further improving clinical care. Digitisation using the NHLS platform was an opportunity to use existing infrastructure which assumed a greater prospect of more immediate scale up of HIV PoCT digitisation as well as PoCT for other conditions.

The Department of Health in the Western Cape consequently implemented a pilot intervention to digitise HIV PoCT results with support from the NHLS transport mechanism and information system in order to establish HIV case-based surveillance. In this study, we evaluated the operational feasibility of this pilot intervention to generate an electronic HIV testing register at a local Community Health Centre (CHC) that would make available individual-level digitised testing data to inform prevention and treatment activities. In this study we adopted a definition of operational feasibility as the ease with which the intervention is supported by the procedures and protocols within the health facility [10]. This study focussed primarily on the recreation of the HIV testing register as a test of operational feasibility and completeness of the digitisation process. Designed to move away from often misleading aggregate data reports to more accurate digitised reporting, the intervention is a critical step in achieving more accurate surveillance of HIV testing and crucial strategic information for moving towards control of the HIV epidemic. In this study we wanted to understand what was operationally possible in a real-world setting. The utility of the intervention, demonstrated by the analysis of individuated data, is reported on elsewhere [20].

In this paper we describe the pilot intervention, present the results of an evaluation, and discuss the operational feasibility of the intervention. We also offer practical implementation reflections into establishing and taking to scale the electronic capture of individual level testing data.

### Setting and intervention

The PoCT intervention evaluated in this study was designed with emphasis on minimal interference to existing workflow, recognising the heavy staff workload and limitations to digitisation within the facility environment.

**Fig. 1:**
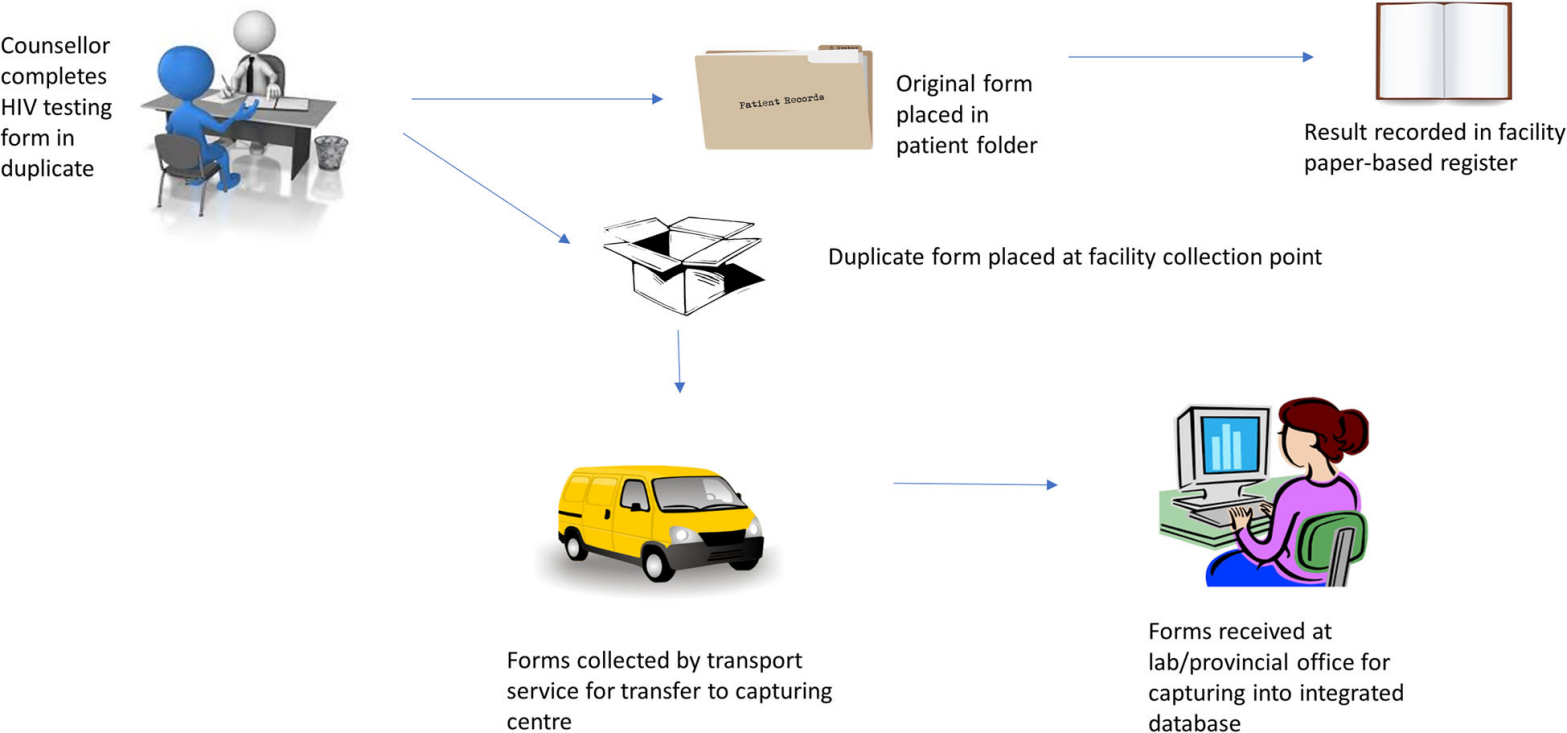
Intervention data flow pathway. Figure generated by Microsoft Powerpoint and freely available for use

The intervention involved transporting carbonated copies of standard HIV testing services (HTS) forms, completed manually by facility staff, to a central point for digitisation and inclusion in the laboratory information system and PHDC (Fig. 1). Routine HTS forms, familiar to staff, were pre-carbonated thereby not requiring the introduction of a new form for digitisation purposes. Pre-carbonation ensured that no additional workload was imposed on staff to duplicate the results for digitisation.

During April 2017, counselling and nursing staff involved in HTS were trained on completion and routing of the carbonated form. Only identifiable patient data (hand-written or on patient labels stuck on the form) and fields relevant to PoCT were captured, including unique HTS form number, demographic data, consent to HIV test, self-reported previous HIV test result, reason for accessing HTS and results of the rapid HIV PoCT. ART data were indicated on forms as free written text and hence not captured due to concerns regarding interpretation errors.

Identifiable data on patient labels included patient name, surname, sex, South African identification number, barcoded folder number, address and telephone number where available. The patient folder number is a computer-generated, barcoded 9-digit number with a coded facility location prefix. This unique folder number is generated by the provincial patient administration system. Patient labels are placed in patient folders on arrival at the reception of most health facilities. These labels are placed on all clinical stationery pertaining to the patient. In order to increase access to HIV testing, patients presenting to health facilities solely for HIV testing are permitted to consult an HIV counsellor directly, without obtaining a folder and patient label at the facility reception. Patient identifying information is then hand-written by HIV counsellors on the PoCT HIV stationery. This reduces waiting times at health facilities for those wanting to test for HIV, thus improving access to testing. Only patients who subsequently test HIV-positive are referred to reception to obtain a folder for further clinical consultation and management.

From May 2017 to June 2018, data were captured from the carbonated copies. It was intended for data to be captured throughout the study period by the NHLS. However, due to logistic delays with the NHLS beyond the study start date, data capturing was divided into two distinct phases which changed the original shape of the intervention.

In Phase I (May to December 2017), forms were transported during regular visits by a project team member and individuated data were captured by an independent trained data capturer on to a provincial database located within the Western Cape Government Health Complex, which was later integrated into the PHDC. Double capturing into the same database was employed for cross-checking to minimise capturing errors; discrepancies were identified and corrected by the data capturer through validation with the carbonated form copy. Data capturing during Phase I was supervised by a project manager with a clinical background (first author). Issues that could not be resolved immediately by the project manager, such as poor identifiers or incomplete forms, were discussed directly with the HIV counsellors and facility management team by the project manager.

In Phase II (January to June 2018), following extensive discussions on pros and cons with NHLS stakeholders, data capturing responsibilities were transferred to laboratory clerks employed by the NHLS. These discussions provided valuable implementation lessons which are discussed as key results of this evaluation. The Phase II approach allowed the use of the routine NHLS transport systems that collect blood and other samples daily from the facility to transport the forms directly to the NHLS. The forms were then captured into the NHLS as per standard operating procedures. Data were checked by a supervisory laboratory technologist who authorised entry into the NHLS information system, with a disclaimer indicating that the results were not verified by NHLS as the tests were conducted by facility staff. Test results were then made available for look-up by clinicians on the NHLS information platform, TrakCare Web Results Viewer. During Phase II, the laboratory project supervisor provided weekly e-mail feedback to the project manager and facility manager on capturing issues. These issues, primarily relating to data quality and completeness, were discussed periodically with the project team (co-authors) and a record of challenges discussed, and solutions developed were documented.

Independent to the intervention, the study facility employed an additional non-governmental organisation (NGO) during Phase II to increase HIV testing at the facility. This change resulted in an increased drive for opportunistic HIV screening in the facility and facility parking lot (community outreach).

## Methods

### Study population

The study population included patients using HTS at Gugulethu CHC, an urban primary care facility in Cape Town. HIV PoCT is offered routinely to pregnant women presenting to the Midwife Obstetric Unit of the CHC, as an outpatient service and during community outreach activities. All patients attending the CHC may voluntarily use HTS or be referred for HTS by health care providers in the CHC. No formal recruitment for the study was required as existing records in routine databases housed by the Department of Health were used for the evaluation.

### Study design

Cross-sectional analyses were conducted on records of all patients at Gugulethu CHC who had an HIV PoCT during the study period. Feasibility research intends to generate evidence on the viability of health system interventions [10]. According to the TELOS framework, this may be categorised into five dimensions, including technology and systems, economic, legal and political and operational and scheduling feasibility [10]. In this study, we focussed on evaluation of the operational feasibility of the pilot intervention, centring on whether the intervention works in practice [10, 21]. Data arising from the intervention were linked to the PHDC using the unique patient identifier and compared with monthly aggregate data from the paper-based register to determine whether electronic registers could be reliably generated from pilot data. Correlation between electronically captured data and paper-based aggregate data was the main indicator of operational feasibility. Percentage linkage to PHDC and completeness of captured variables were secondary indicators of operational feasibility.

### Data analysis

Data were analysed using Microsoft Excel and Stata 14 (Stata Corp, USA). Personal data obtained from the PHDC were de-identified before release for analyses.

HIV outcome was calculated electronically using the National Department of Health HIV testing algorithm, in which serial HIV rapid testing is required [22]. If the initial screening rapid test is positive, this is followed by a different confirmatory rapid test. If both rapid tests are positive, a diagnosis of HIV is made. The rapid test algorithm is repeated immediately if initial results are discordant. If discordance persists, an ELISA test for laboratory confirmation is required [22].

Once validated, captured data were exported and linked to existing patient records in the Patient Master Index through PHDC, using unique patient folder numbers. Where folder numbers were unavailable or illegible, linkage was attempted using combinations of other patient identifiers such as name, surname and date of birth. Unlinked forms were those with limited identifiers or that could not be definitively linked to a single patient with the available information [23].

Data arising from the intervention were compared with monthly aggregate data from the paper-based register submitted to the District Health Management Information System equivalent in the Province, a web-based aggregate data portal named SINJANI. Electronically-captured data were filtered according to month to generate monthly electronic HIV testing registers for comparison with routine monthly aggregate data. Key reporting elements in the 2018 official HTS register as reported on SINJANI were used for more granular comparisons of aggregate data from July 2017 to December 2017. Correlation coefficients were calculated to quantify the correlation between the two monthly datasets. Pearson’s correlation coefficient (r_p_) was used for normally distributed variables and Spearman’s coefficient (r_s_) was used for non-parametric variables. Of the 49 reporting elements in the official 2018 HTS register, 13 were used for comparisons (Table 1). The register comprises of 4 broad categories viz. 18 elements specific to antenatal care (ANC), 11 elements specific to children under the age of 15 years, 13 elements pertaining to all patients, including ANC and 7 elements excluding ANC. Selected elements represented these categories, however those with respect to children 15 years and under were excluded, as Gugulethu CHC does not cater specifically for children. Ethical approval for the evaluation was obtained from the University of Cape Town Human Research Ethics Committee (HREC 198/2018).

**Table 1:** Selected official 2018 provincial indicator dataset reporting elements and definitions

Reporting element	Definition
Total HIV test client accept test (incl. ANC)	Any client who accepted an HIV test including antenatal clients
Clients PRE-TEST counselled for HCT (incl. ANC)	Any client counselled for HIV test including antenatal clients
Antenatal client counselled for HIV testing - 15 to 24 years	Antenatal client aged 15–24 years counselled for HIV testing
Antenatal client counselled for HIV testing - 25 years and older	Antenatal client aged 25 years and older counselled for HIV testing
Accept testing - PMTCT initial test	Antenatal client who was tested for the first time during her current pregnancy
Accept testing - PMTCT repeat test at 20 weeks	Antenatal clients who tested negative for HIV during an earlier antenatal visit and were re-tested for HIV at 20 weeks during the pregnancy
Accept testing - PMTCT repeat test at 32 weeks	Antenatal clients who tested negative for HIV during an earlier antenatal visit and were re-tested for HIV at 32 weeks during the pregnancy
HIV positive client category - PMTCT initial test	Antenatal clients who tested positive for the first HIV test done during the current pregnancy
Antenatal client eligible for ART	Antenatal client eligible for ART as per current guidelines
Known HIV positive antenatal client	Antenatal clients with known HIV positive status. In the absence of documented proof, verbal confirmation of HIV status is acceptable
HIV test 15 years and older (excl. ANC)	Any client aged 15 years and older tested for HIV excluding antenatal clients
HIV test client 15–24 years (excl. ANC) accept test	Any client aged 15–24 years tested for HIV excluding antenatal clients
HIV PRE-TEST counselled client (excl. ANC)	Any client counselled for HIV test excluding antenatal clients

### Management team reflections and observations while managing implementation

To support the presentation of the evaluation results, the project management team (co-authors of the paper) in the Provincial Department of Health, who function as participant observers in managing the overall implementation of the intervention, have pooled together their personal reflections to generate implementation lessons. These personal reflections were captured in weekly logs including personal notes and reflections on stakeholder meetings, weekly face to face correspondence with project staff and e-mails. The first author consolidated these personal reflections and grouped them thematically into lessons. These were reviewed by the members of the project management team. We acknowledge the limitations of one perspective on implementation, as the project management team brings only one viewpoint on successes and challenges. Other local level actors, including the CHC facility manager and data capturers in both phases, for example, may have different or additional viewpoints on successes and challenges.

## Results

### Key evaluation results

From May 2017 to June 2018, 11,337 HTS forms were digitised. These were linked to the PHDC, with 70% (7954) being captured during Phase I and 30% (3383) during Phase II. In total, 137 forms were excluded from aggregate analyses due to incorrectly entered dates of test. Overall, 97% linkage to the PHDC was achieved. Linkage of forms to the PHDC was 96% (7667/7954) in Phase I and 98% (3315/3383) in Phase II. Completeness of variables exceeded 97% apart from HIV testing outcome (86%) and reason for accessing service (73.9%).

**Fig. 2:**
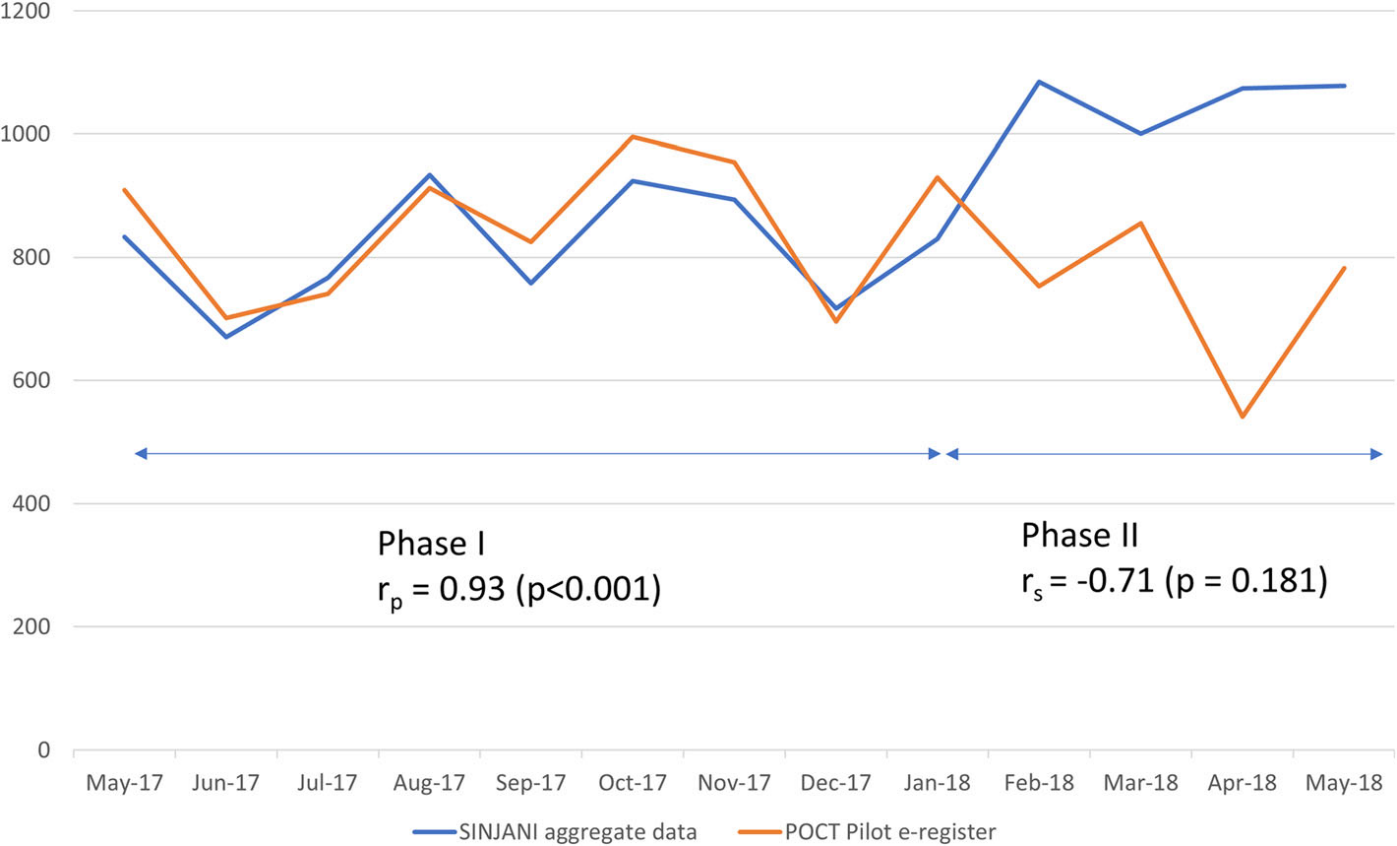
Comparison of total number of patients accepting an HIV test (including antenatal testing)

A comparison of aggregate data submitted to SINJANI, independent of the pilot, and monthly data collected during Phase I, showed a high correlation (*r* = 0.93, *p* < 0.001). In Phase II, correlation between the two datasets was lower (*r* = − 0.71, *p* = 0.1812). Figure 2 illustrates the differences between the two phases. The divergence between the two phases present an opportunity for us, the project management team, to reflect on the implementation opportunities and challenges that may have influenced these differences.

**Fig. 3:**
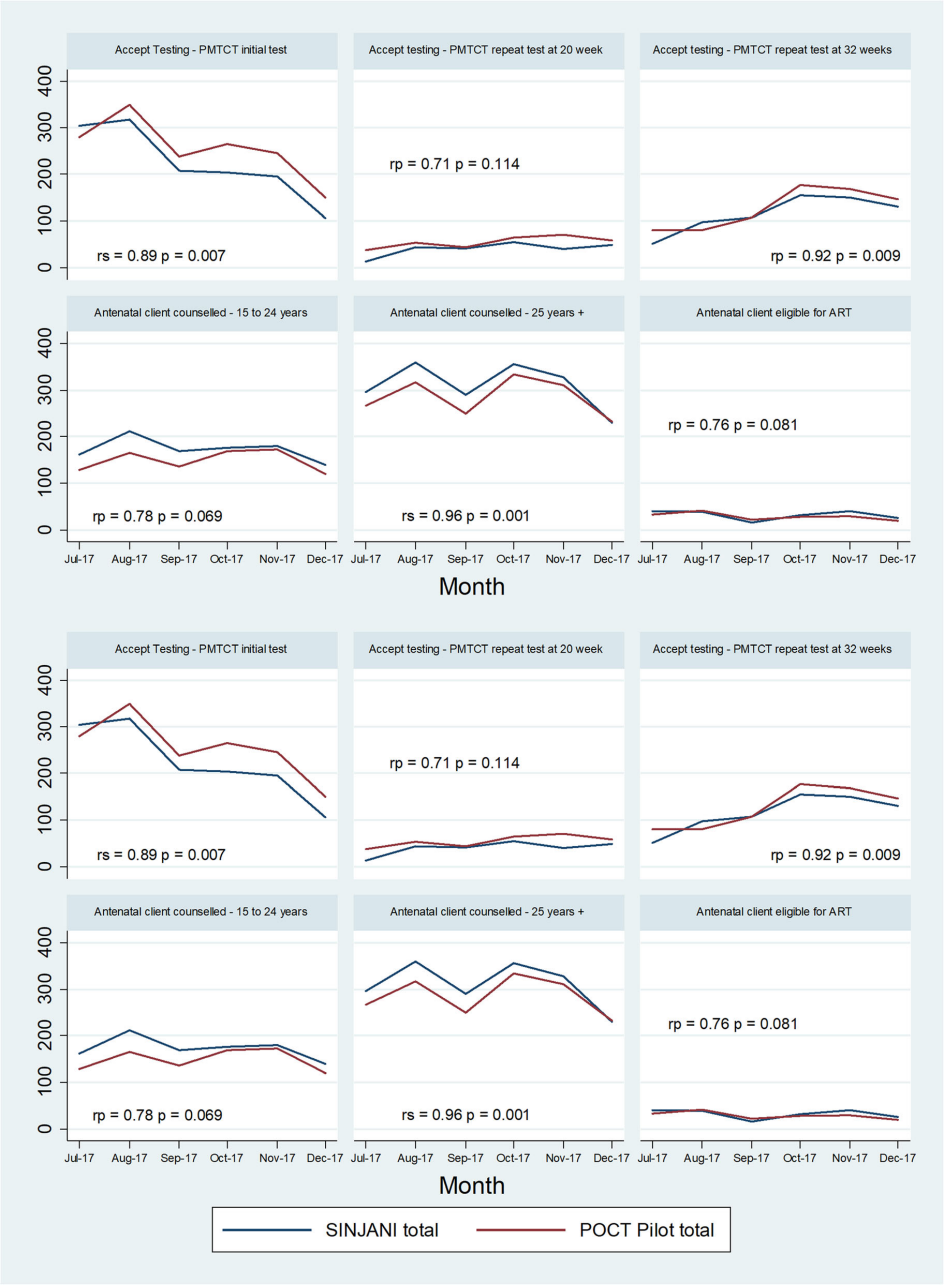
Comparison of HIV testing register reporting elements, July to December 2017

Key reporting elements from the routine HIV testing register extracted from SINJANI (Table 1) were further compared to pilot data for the period July to December 2017, when Phase I was well established. Figure 3 demonstrates closely correlated trends between the official aggregate totals reported from the paper-based HTS registers, and the aggregated digitised data from the PoCT-digitisation intervention.

### Key implementation lessons

While implementing the pilot intervention, there were a number of challenges and opportunities during the digitisation process. Key challenges included stakeholder concerns around data quality issues and logistics. Some of these challenges were mitigated over time through a series of helpful solutions, while others remained unresolved.

#### Stakeholder concerns

Before commencing the intervention, several stakeholder engagements took place in the form of face-to-face meetings and via e-mail. A key concern expressed by the NHLS was the perceived lack of quality assurance for PoCT conducted by clinical staff who lacked laboratory training in quality control. The NHLS has strict quality control measures in place for testing which is different from the daily practices at facility level. Furthermore, the capturing of hand-written results from clinical stationery, did not always comply with the strict accreditation standards upheld by NHLS. The NHLS was cautious about issuing results for assays they had not performed, and which were not subject to standard laboratory quality controls. The complexity of the routine clinical form used in the clinical environment which had multiple fields with clinically-relevant information, was also identified by NHLS as a barrier to capturing in a laboratory setting, compared to the structured laboratory-request forms used for requesting tests conducted by them. Additionally, the NHLS was concerned that the presentation of HIV PoCT results on the web viewer may cause confusion in clinical settings where other tests may be discordant with the PoCT, particularly given the quality concerns. In order to address these concerns, a clear disclaimer was used on the web look-up to waiver NHLS responsibility for the results and circumvent reputational risk. PoCT results for each rapid test (both screening and confirmatory test) performed on a patient were presented exactly as captured by the counsellor, without any interpretation of the final HIV outcome. This would allow clinicians to exercise clinical judgement when reviewing PoCT results in conjunction with other laboratory results.

Confidentiality of PoCT information on route to NHLS and during capturing by laboratory clerks was another concern raised by NHLS. Following extensive discussion, it was acknowledged that whilst HTS forms do contain sensitive patient information, the information is similar in nature to clinical information written on standard laboratory request forms that are routinely transported from facilities to NHLS and captured by laboratory clerks. Many laboratory request forms contain HIV status information as required clinical background for laboratory tests. These are transported routinely in laboratory specimen bags. The pilot intervention therefore does not confer any additional risk when compared to standard laboratory transport practices. However, in order to ensure confidentiality of HTS forms during transport in both phases, forms were sealed in envelopes (instead of transparent specimen bags) in the counsellors’ offices before transferring to capturing sites.

There were concerns that the proposed intervention would increase NHLS workload for data clerks and technologists and that any such increases would not be adequately compensated. After 10 months of discussions on the pros and cons of the intervention, at both the national and provincial level (February to December 2017), NHLS agreed to participate in Phase II of the pilot. Capturing processes were handed over to the NHLS at a higher cost than Phase I and were based on internal NHLS assessments of the fixed costs associated with specimen transport and registration.

#### Data quality

Having commenced the pilot, it became clear that poorly legible and incorrectly completed forms were an issue in both phases of the pilot. Incorrectly completed forms included those in which identifiers were poorly completed in the absence of patient labels, incomplete consent fields and testing results entered in incorrect fields. Inconsistent entries were also identified; for example, free text indicating that a patient testing HIV negative is on ART. Regular face-to-face feedback was provided to counsellors and facility management in order to address these data quality issues. While some issues were resolved during Phase I, many of these data quality issues persisted in both phases and worsened in Phase II when more counsellors provided HTS at the facility. Issues specific to the printing of forms included legibility of carbonated copies, uniformity of carbonation and erroneous printing of duplicate form numbers. These issues were discussed with the printers and form books modified accordingly. Incorrect application of the testing algorithm was identified as a major testing and data quality issue which would impact both paper-based and electronic registers. In order to mitigate this data quality issue in study analyses, only electronically calculated HIV outcome was analysed, based on the screening and confirmatory test results.

During Phase II, resolution of the above-mentioned form-specific issues were not addressed efficiently, as in Phase I, due to limited direct communication between NHLS and facility management and staff. Furthermore, poorly legible forms and incomplete forms were immediately rejected for capturing due to the strict accreditation protocols followed by NHLS.

The introduction of the additional NGO at the facility during Phase II resulted in new HTS counsellors who required training, and among whom there was a high staff turnover. This in turn appears to have resulted in more data quality issues and form rejections by NHLS. Furthermore, the increase in outreach testing resulted in more patients without computer-generated patient labels and unique folder numbers. The hand-written patient identifiers used for these patients were unacceptable to the NHLS. Forms without patient labels frequently lacked a location code, another accreditation requirement. Other challenges included the use of computer-generated temporary numbers due to technical issues on the electronic administrative platform, which were not recognised by the NHLS and poor-quality printing of patient labels impacting legibility. These were also rejected by NHLS. Some counsellors erroneously used computer-generated patient labels from other sites that patients occasionally kept with the patient-held case record. These labels contained location codes of facilities external to the pilot site and hence were rejected by the NHLS.

As a response to these challenges, a direct weekly e-mail communication chain between NHLS and the facility manager was instituted during Phase II so that both parties could better understand and address form-related concerns and facility-related factors such as training of HIV counsellors, patient registration issues and others. Despite these efforts, both parties had limited direct communication, leaving many issues unresolved during Phase II. The digitisation process, however, was able to identify errors in HIV testing and form completion which has the potential to meaningfully contribute towards clinical governance by identifying areas for further staff training and support.

#### Logistical issues

Several logistical issues relating to routing and transport of the forms were identified and addressed on an ongoing basis. These issues were the unavailability of envelopes for confidential transport, poor labelling of envelopes, delays in the daily routing of forms and incorrect routing of original forms, instead of the carbonated copy. Although the NHLS requested that forms were sent to them daily for a consistent workload, counsellors often batched forms over several days due to delays in entry into the paper-based register. This also limited the ability of the NHLS to accommodate the extra workload. The issues were addressed by providing pre-labelled envelopes to the facility, regular monitoring of form and envelope stock availability by the project manager and direct communication of issues with facility staff by the project manager. The use of pre-labelled envelopes for matters unrelated to the pilot was difficult to manage remotely as the project manager was not based in the facility. Adopting an implementation process of identifying and addressing challenges as they arose by the project management team in consultation with those working on the ground led to success during Phase I.

#### Feasibility of intervention

During the pilot (Phase I and II combined) we achieved almost universal (97%) linkage in the PHDC and variable completeness (most variables > 97%). High levels of data completeness, data linkage and, most importantly, correlation between the aggregated data and Phase I data, indicates that it is operationally feasible to replace manual testing registers with electronic registers in the Western Cape. A comparison of specific reporting elements from routine reports during Phase I revealed marginal differences, confirming that electronic registers may efficiently replace paper-based registers at a more granular level. We have included implementation lessons in this paper that supported that success in Phase I, including a dedicated project manager, regular discussions with the project management team on challenges and the following up with solutions.

Although Phase I was able to closely replicate the paper-based register, notable divergence occurred in Phase II due to the high number of form rejections. Only forms with clear identifiers were captured during Phase II, resulting in the observed higher linkage to the PHDC. During Phase I, however, all available identifiers including hand-written identifiers, temporary numbers and identifiers on labels from other facilities were captured as best as possible for potential linkage to the PHDC. These different approaches may represent differing stakeholder priorities during the two phases. During Phase I, the Western Cape Department of Health captured and linked all validated forms through the PHDC, as any data indicative of an encounter with HTS services can considerably enrich monitoring and surveillance of the PHDC HIV treatment cascade, which is intended to directly impact individual patient care. The provincial platform had high interest in the success of the pilot and the outcome of the intervention was of primary focus. Phase II was more process-driven as adherence to accreditation criteria and quality control was paramount to the NHLS.

During Phase I, data quality issues were better addressed with clinical input and direct communication and feedback between facility staff, counsellors and data capturer. This communication was heavily driven by the project manager who visited the site weekly. Additionally, double capturing of data allowed identification and resolution of capturing errors. Without a regular feedback mechanism and the clinical and contextual understanding in Phase II, issues remained unresolved leading to greater numbers of form rejections and incomplete electronic registers. For the NHLS, central laboratory test results are requested by a named clinician, therefore there is an inherent feedback loop where incorrect testing procedures or specimen request entries will impact the availability of results needed by the clinician. In the Phase II setting, however, the counsellors were not involved in the feedback loop, as the captured data are not required by the counsellors and a form rejection does not affect the relevant counsellor directly. Direct involvement of facility management may thus increase accountability of individual counsellors who report to the facility manager.

## Discussion

### Operational feasibility

Phase I was successful in replicating the manual register. Phase II showed that the capturing mechanism worked but cannot replicate the paper-based register yet. Our study shows that the pilot intervention introduced by the Western Cape Department of Health to digitise PoCT HIV test results could successfully replace paper-based HIV registers. However, we note that there are a number of implementation factors that need to be proactively managed in context to achieve success. Key reflections from the project management team to inform a sustainable scale-up strategy for an electronic HIV testing register in a high diagnosing setting include the importance of regular communication between central data managers and facility staff, the value of clinical and operational insight when designing and implementing health information system interventions and the need for detailed stakeholder engagement prior to implementation. Also, in Phase I, a project manager being available at all times to address challenges and propose solutions in real time played an important role.

This intervention, if scaled up, can contribute to case-based surveillance and improved monitoring of performance towards the 90–90-90 targets. Importantly, this approach can also provide actionable individuated patient data which can directly improve the care of individual patients by identifying those who do not link to ongoing care. Furthermore, the study demonstrates the value of the intervention in strengthening clinical governance and its potential to improve operational efficiency at health facilities by decreasing workload required to maintain paper-based registers and enhancing clinical information available for consultations, should electronic registers be implemented.

### Stakeholder engagement

A highly consultative, bottom-up approach was used in the design of the intervention, where the needs of frontline staff members were considered and incorporated in the development of the intervention. A key characteristic of the intervention was its minimal disruption to routine facility practices. Although this bottom-up approach was welcomed by facility staff, it conflicted with central laboratory implementation methods where a predominantly top-down approach is employed. The latter approach assumes a well-resourced environment where implementation is a straightforward technical process guided by strict criteria and standards [24]. Although both have their advantages and disadvantages, a fine balance is required for successful implementation and scale-up [25–27]. Balthuis et al. clearly describe the importance of considering all stakeholder perspectives when planning scale-up of an intervention [28]. In this study, the discord in stakeholder approaches resulted in the two distinct phases of data capturing and notably different findings during the phases.

The divergence between Phase I and Phase II may be closely linked to NHLS concerns relating to quality assurance, differentiating PoCT results from central laboratory tests and adherence to accreditation criteria. These are all well-documented concerns [18, 19]. Studies have shown an increase in pre-analytical errors with PoCT compared to central laboratory testing, resulting in a greater potential for false negative and false positive results [18, 19]. Recording of PoCT results in patient records were found to have far more errors than recording of central laboratory tests [19]. Given the data quality issues identified during the pilot, concerns regarding the quality of PoCT are well-founded and may be addressed through more rigorous training. Interventions such as these may have been better received when driven by stakeholders with expertise in central laboratory processes. This notion of professional exclusivity is well-described in literature as a health system-related barrier to adoption of PoCT strategies [18]. Differing workplace priorities and routine practices contribute significantly to these different stakeholder approaches [19]. Strict adherence to high standards is paramount to uphold the quality of services. This ardent and commendable commitment to quality service provision was evident in the NHLS approach to the intervention. In practice, different stakeholders may have different goals and standards which can cause friction during implementation. Issues related to PoCT, described above, exist regardless of whether paper-based or electronic information systems are used. However, digitisation and linkage into electronic information systems may more readily identify quality control and other systemic PoCT issues for targeted remedial action, and therefore enable better collaboration and trust between facility and central laboratory stakeholders.

### Champions in healthcare

A key success of Phase I was the direct communication and feedback mechanism that was introduced between data capturers, situated at the Provincial Department of Health complex, and facility staff. This was driven by the project manager, who adopted the role of an implementation “champion” and liaised between different actors. The champion construct is defined as an “implementation-related role occupied by people who are internal to an organisation, generally have an intrinsic interest and commitment to implementing a change; work diligently and relentlessly to drive implementation forward; are enthusiastic, dynamic, energetic, personable, and persistent; and have strength of conviction” [29].

The appointment of an intervention champion has previously been shown to positively impact on healthcare-related implementation in various clinical and organisation roles [29, 30]. During Phase II, the role of champion at the Provincial Department of Health was diminished as capturing took place at NHLS. Regular supervision of data capturing was undertaken by NHLS rather than the project manager; a move that represents a more feasible strategy for future scale-up using the NHLS platform. Although efforts were made to set up a communication mechanism between NHLS and the facility, these were largely unsuccessful due to competing priorities and limited direct interest in the project. Communication mechanisms may have benefited from an active champion in Phase II.

Our findings concur with a number of other studies on the value of champions in healthcare [29]. However, champions alone are insufficient to ensure implementation success as other contextual factors also require consideration such as stakeholder buy-in, training needs and resource availability [29]. Inclusion of champions may strengthen sustainability of a new intervention; however, this approach may notably increase the costs of scale-up as further human resources may be required in an already over-burdened health care environment.

### Implementation challenges

Data quality issues, including illegible, incomplete or incorrect data entry, were a concern during implementation and reflect poor clinical record keeping more generally. Completeness and accuracy of routine data entry is imperative for a functional health information system [14].

The intervention was designed to have minimal impact on routine facility workflow, as a commonly held perception among staff at facility level is that improved data collection requires increased administrative workload for staff. Despite these considerations, the poor quality of routine data is an ongoing challenge in many settings [13, 31]. Reasons for poor quality of routine data include lack of accountability, limited understanding on the use of data, conflicting priorities in clinical work environments and involvement of multiple individuals in data entry [13].

Of interest, reason for accessing service, which was generally poorly captured, was better captured in antenatal services where reporting elements specific to this variable are required in the HTS register. This suggests that form completion may be driven by an awareness of what the data are used for, and that providing routine feedback to persons collecting the data (rarely experienced) that makes clear how these data are used for individual and population gain may result in improved data collection [13].

The value of an intervention such as ours cannot be demonstrated fully until the process is adequately implemented over time. This issue was exacerbated in Phase II due to the scale-up of testing services and high turnover in counselling staff. Minimal staff turnover is a recognised advantage in health system strengthening interventions due to limited training needs [32]. Without routine and on-going training of new facility staff, the benefits of increased testing are limited due to errors in testing, interpretation of results and data entry [18, 19]. Line management hierarchies are also complex: the HIV counselling staff are not direct employees of the facility or the Department of Health, but members of NGOs contracted by the province to provide these services. In implementing our intervention, it would be preferable to standardise training and registration of HIV counsellors centrally. In the current setting, the drive to meet 90–90-90 targets may result in unintended negative consequences such as retesting of known HIV-positive patients in an effort to achieve testing targets set by the Department of Health.

A formal analysis of costing was not conducted in this evaluation, however resourcing will have a notable impact on scale-up. The high cost incurred during Phase II is a notable challenge to province-wide scale up. Subramanian et al. note the need for stakeholder engagement, strong leadership and realistic financing for scale-up [33]. Despite initial assumptions of the Phase II model being more pragmatic, scale-up using the Phase I model appears more operationally feasible and less costly, but will still require significant resource investments for stationery, transport and capturing on a wider scale as well as rigorous staff training and involvement of facility management. This may, however, result in higher costs due to inefficiencies in maintaining a parallel provincial system and the need for a champion to drive the process forward. From the central laboratory perspective, the intervention is onerous, poorly adherent to laboratory standards and of limited value. Higher level collaboration is required to streamline the intervention if it is to be supported by NHLS.

### Advantages of digitisation

The eventual replacement of manual testing registers with electronic registers has several advantages. The digitisation of individuated data provides significant gains in population-level case-based surveillance [3], while also enriching available patient information at clinical consultations. Further surveillance analyses, beyond the standard reporting elements, is possible through digitisation and linkage to individuated data. The exclusive use of electronic registers has the potential to significantly reduce the workload of clinical staff tasked with maintaining large paper-based registers. A complete replacement of paper-based registers with electronic registers can create more incentive to implement the intervention successfully while also tangibly decreasing the workload of maintaining paper-based systems during the intervention and thus enhancing feasibility of the intervention.

The intervention also uncovered various training gaps in routine clinical record-keeping and the HIV testing protocol and algorithm. Improved training, support and management of frontline healthcare staff, including externally contracted staff, is essential to ensure quality of patient care and data integrity. Digitisation may also provide more accurate workload estimates for counselling staff, thus facilitating evidence-based resource allocation within facilities and improved operational efficiency [5, 14].

The intervention may further demonstrate the contribution and inefficiencies of massive testing campaigns to achieve the first 90 of the 90–90-90 strategy. Although not detailed in this study, the added contribution of individuated data within the electronic register, may further contribute to improving linkage to care through active surveillance and improved monitoring of the second and third 90 within the 90–90-90 strategy.

### Looking to the future in South Africa

Since the PHDC integrates various data sources, including laboratory data, there may be no added value in digitisation on the NHLS platform as long as the PoCT results are accessible to clinicians in a similar way to central laboratory results look-up. Despite the potential for false negative or positive results due to data quality issues, digitisation of PoCT will still confirm engagement at HTS services for PoCT, which is valuable clinical information for case-based surveillance and individual patient history-taking.

The presence of facility-based laboratories and laboratory staff within facilities may be an alternative implementation model, preferable from the laboratory perspective [18, 19]. Similarly, the use of PoCT devices or mobile devices with direct electronic capturing are useful options, particularly with current approaches to expand testing to outreach sites, outside traditional facilities. Direct digital capture would also eliminate manual entry and subsequent challenges related to poor handwriting. These options are, however, notably resource intensive, with limited scope for scale up currently in both clinical and community settings [19]. Capturing of HTS forms at the facility, either by counselling staff or by dedicated data capturing staff may also be considered, thus eliminating the need to route forms offsite. These options again rely on the availability of infrastructure and networking capabilities in all clinical environments as well as additional trained staff members, given the heavy workloads of facility-based staff members. Further analysis of the feasibility and cost implications of implementing such models is required.

This pilot clearly illustrates the importance of balancing the needs and values of different stakeholders when developing and implementing an intervention. Appropriate stakeholder (health care facility management and staff, NHLS, NGO and operational staff) ownership of the intervention is needed in order to facilitate better communication and feedback. A more detailed stakeholder analysis with consideration of preconceived stakeholder attitudes is thus imperative before considering scale-up [34, 35]. Furthermore, the challenges identified are reflective of broader health-system, operational and economic challenges in resource-limited settings [3, 14].

### Limitations

We recognise that our study has several limitations. The findings of this study are context specific. Given that health information systems may vary significantly within districts, provinces and countries, implementation strategies may need to be adapted according to the context [33]. The focus of the study was operational feasibility, thus other dimensions of feasibility have not been explored and may impact scale-up. Similarly, economic viability of the intervention was not formally evaluated. The validity of the data captured in the routine aggregate SINJANI reports was not explored in this study and may be subject to the known biases of routine data [13]. A key limitation of the implementation lessons is that it only reflects the views of the provincial implementation management team. We did however send drafts of these lessons for member checking to the NHLS project manager, who was one of the key implementing partners. Drafts were approved by the NHLS project manager as adequately representative of their implementation experience. Evaluation of the roll out of a systems intervention of this nature would no doubt benefit from a formal implementation analysis including multiple stakeholder perspectives.

## Conclusions

In Phase I, this study demonstrated the operational feasibility of rapidly digitising HIV PoCT data to contribute to consolidated actionable person-level longitudinal HIV case-based surveillance without compromising currently collected aggregate indicator data. The implementation model of minimally changing current practices through simple interventions such as pre-carbonated forms and utilising existing transport mechanisms for specimens to centralise forms to a high throughput capture environment, was demonstrated to be readily achievable. Routing the data through the NHLS had unanticipated challenges and costs and may require further improvements in both PoCT platforms as well as clerical and administrative systems before the approach reaches its full potential. There are a number of options for HIV (and other) PoCT digitisation ranging from the Phase I model in this study (centralisation of duplicate forms) to a variety of facility-based and tester-based solutions, the optimal choice of which is likely highly context-specific.

## Data Availability

The dataset supporting the conclusions of this article is available in the Provincial Health Data Centre, Western Cape Provincial Department of Health. Release of these data to a public domain would violate the Data Access Policy of the Western Cape Department of Health. Data requests may be sent to the Western Cape Provincial Department of Health: Phdc.Pgwc@westerncape.gov.za

## Abbreviations

ANC: Antenatal care
ART: Antiretroviral treatment
CHC: Community Health Centre
ELISA: Enzyme-linked immunosorbent assay
HIV: Human immunodeficiency virus
HTS: HIV testing services
NHLS: National Health Laboratory Services
PHDC: Provincial Health Data Centre
PoCT: Point of care test
UNAIDS: United Nation’s programme on HIV/AIDS
WHO: World Health Organization
